# Adhesion molecule cross‐linking and cytokine exposure modulate IgE‐ and non‐IgE‐dependent basophil activation

**DOI:** 10.1111/imm.13268

**Published:** 2020-10-29

**Authors:** Frida Kalm, Ladan Mansouri, Aman Russom, Joachim Lundahl, Anna Nopp

**Affiliations:** ^1^ Department of Clinical Science and Education Karolinska Institutet Södersjukhuset Stockholm Sweden; ^2^ Division of Nanobiotechnology Department of Protein Sciences Science for Life Laboratory KTH Royal Institute of Technology Stockholm Sweden; ^3^ Sachs' Children and Youth Hospital Södersjukhuset Stockholm Sweden

**Keywords:** CD203c, CD62L, degranulation, miBAT

## Abstract

Basophils are known for their role in allergic inflammation, which makes them suitable targets in allergy diagnostics such as the basophil activation test (BAT) and the microfluidic immunoaffinity basophil activation test (miBAT). Beside their role in allergy, basophils have an immune modulatory role in both innate immunity and adaptive immunity. To accomplish this mission, basophils depend on the capability to migrate from blood to extravascular tissues, which includes interactions with endothelial cells, extracellular matrix and soluble mediators. Their receptor repertoire is well known, but less is known how these receptor–ligand interactions impact the degranulation process and the responsiveness to subsequent activation. As the consequences of these interactions are crucial to fully appreciate the role of basophils in immune modulation and to enable optimization of the miBAT, we explored how basophil activation status is regulated by cytokines and cross‐linking of adhesion molecules. The expression of adhesion molecules and activation markers on basophils from healthy blood donors was analysed by flow cytometry. Cross‐linking of CD203c, CD62L, CD11b and CD49d induced a significant upregulation of CD63 and CD203c. To mimic in vivo conditions, valid also for miBAT, CD62L and CD49d were cross‐linked followed by IgE‐dependent activation (anti‐IgE), which caused a reduced CD63 expression compared with anti‐IgE activation only. IL‐3 and IL‐33 priming caused increased CD63 expression after IgE‐independent activation (fMLP). Together, our data suggest that mechanisms operational both in the microfluidic chip and in vivo during basophil adhesion may impact basophil anaphylactic and piecemeal degranulation procedures and hence their immune regulatory function.

## Graphical abstract

Basophil adhesion and activation are important steps for the basophil immune modulatory functions and can be investigated with flow cytometry and microfluidics (miBAT). Cross‐linking of CD203c and the adhesion molecule CD62L, which mimics both adhesion in vivo and capture in miBAT, has a regulator effect on IgE‐mediated (anti‐IgE) basophil degranulation, by reducing the CD63 expression compared with anti‐IgE activation only. In addition, IL‐3 and IL‐33 enhance the CD63 expression on basophils after IgE‐independent (fMLP) activation compared with fMLP activation only.

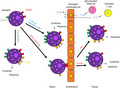

**Abbreviations**
BAT: basophil activation test
CD: cluster of differentiation
fMLP: N‐formyl‐methionyl‐leucyl‐phenylalanine
IL: interleukin
MFI: mean fluorescence intensity
miBAT: microfluidic immunoaffinity basophil activation test
PBS: phosphate‐buffered saline
PDMS: polydimethylsiloxane
RBC: red blood cell
RPMI: Roswell Park Memorial Institute
RT: room temperature
VLA‐4: very late antigen 4

## INTRODUCTION

Basophils are rare granulocytes circulating in blood, playing a variety of roles in inflammation and immunomodulation. Basophils are involved in the protection against parasites such as helminths,^1^ drive T helper 2 cell differentiation by secreting interleukin (IL)‐4, activate B‐cell production of IgE and are implicated in allergic diseases in general.^2^ To accomplish these functions, the basophil heavily depends on the capability to migrate from blood to different extravascular tissues, for example lungs and lymphoid tissues.^3^ Cytokines and chemokines from the site of inflammation attract the basophil to the endothelium. The basophil rolls on the endothelium surface using selectins followed by an upregulation of integrins, which allow the basophil to firmly adhere to the endothelium. The basophil will then transmigrate through the endothelium to the tissue.^3^

Basophils can be activated via two distinct mechanisms, IgE‐dependent and IgE‐independent activation. Allergen cross‐linking of IgE bound to the surface of the basophil activates the cell during allergic reactions in an IgE‐dependent manner, while the bacterial peptides, for example N‐formyl‐methionyl‐leucyl‐phenylalanine (fMLP), activate basophils in an IgE‐independent manner.^4^ Upon activation, basophils release preformed mediators by two processes, that is anaphylactic degranulation and piecemeal degranulation. In anaphylactic degranulation, histamine, IL‐4 and leukotriene C4 are released and CD63 is exposed on the basophil surface. In piecemeal degranulation, only a very low concentration or no histamine and CD63 are released or exposed, but both anaphylactic and piecemeal degranulation upregulate CD203c on the basophil surface.^5^, ^6^

Basophils are of interest in allergy diagnostics because of their role in allergy and easy accessibility in blood. The basophil activation test (BAT) is an in vitro basophil allergen challenge test analysed by flow cytometry and used in allergy diagnostics. The test is clinically utilized and can be used to indicate how sensitive a patient is to an allergen.^7^ The technique measures the expression of surface markers, for example CD203c and CD63 on basophils following allergen exposure.^8^, ^9^ To further improve the applicability of this method and to allow for a future point‐of‐care testing, we have previously developed a microfluidic immunoaffinity basophil activation test (miBAT) technique that can capture basophils in a microfluidic chip^10^ followed by basophil activation.^11^ However, a challenge related to the miBAT is the background expression of CD63 in non‐activated chip‐captured basophils, which results in a more narrow analysis range.^11^ To enable further improvement of the technique, this matter needs to be addressed.

Several plausible mechanisms exist to explain the background activation of captured basophils in the microfluidic chip, that is activation caused without engagement of the IgE receptor. Interactions between the cell and the capture antibodies coating the chip surface, binding to absorbed proteins such as fibronectin and interaction with soluble factors such as cytokines in the plasma are some possible mechanisms. CD203c is used for basophil‐specific capture on the chip surface, and cross‐linking of this marker or other cellular adhesion molecules could potentially cause basophil activation. The adhesion process involves L‐selectin (CD62L), CR3 (CD11b, CD18) and VLA‐4 (CD49d, CD29), where fibronectin is a ligand for both CR3 and VLA‐4.^12^ CD62L has previously been shown to have a stable expression on basophils upon activation,^13^ in contrast to neutrophils and monocytes, but its function is not yet fully established. All three adhesion markers are involved in different steps in basophil rolling, adhesion and transmigration in vivo,^14^ and these events are applicable also in the microfluidic chip.

Interaction with present cytokines in plasma, such as IL‐3 and IL‐33, could be an additional explanation for the basophil background activation in the miBAT. These cytokines are also key factors in the in vivo process of basophil adhesion, transmigration and activation. IL‐3 is a major growth factor for basophils and is important for both development and survival. IL‐3 can be released by basophils and facilitates an autocrine IL‐3 response.^15^ IL‐33 is an alarmin secreted by endothelial and epithelial cells during, for example, infection^16^ and in asthma^17^, ^18^ and can activate basophils into secreting IL‐4 in the presence of IL‐3. Both cytokines are also involved in the recruitment of basophils into the site of inflammation.^19^

Given that the basophil activation status is of critical significance both in the miBAT technique and during the in vivo recruitment process, we aimed to investigate the impact of basophil interaction with capture antibodies, adhesion molecule ligands and keynote chemokines on the degranulation process. We hypothesized that adhesion and interaction with chemokines, operational both in vivo and in miBAT, affect basic basophil functions such as the responsiveness to subsequent activation.

## MATERIALS AND METHODS

### Study population

Blood donors (18–65 years old) were recruited from blood banks in Stockholm. Informed consent was obtained from all study participants.

The study was approved by the ethics committee in Stockholm, Sweden (2014/1630‐31/4).

### Blood sampling

Venous blood samples (9 ml) from healthy donors (blood bank, Stockholm, Sweden) were collected in sodium heparin vacutainer tubes (Vacutainer, Becton Dickinson, USA) and stored at 4° pending analysis. All experiments were analysed within 4 h from blood sampling.

### Flow cytometric analysis of surface markers before and after passing through a microfluidic chip

The effect on surface marker expression was investigated on cells before and after passage through a microfluidic chip coated with 1% bovine serum albumin. Two hundred µl whole blood was flown through two separate microfluidic chips at 3 µl/min,^10^ and the blood was collected at the outlets. One hundred 100 µl blood, from before and after passage through the microfluidic chips, was put on ice and incubated with antibodies of the recommended volumes (5 µl for all antibodies except anti‐CD62L for which 2 µl was used). The basophil panel included the following: anti‐CD203c‐PE (NP4D6, BioLegend, San Diego, CA, USA), anti‐CD63‐FITC (H5C6, BioLegend), anti‐CD62L‐Alexa Fluor 700 (DREG‐56, BioLegend), anti‐CD11b‐PE/Dazzle 594 (ICRF44, BioLegend), anti‐CD49d‐APC (9F10, BioLegend) and anti‐CD193‐PerCP/Cy5.5 (5E8, BioLegend). The neutrophil panel included the following: anti‐CD15‐PE (HI98, Thermo Fisher Scientific, Waltham, MA, USA), anti‐CD16‐FITC (3G8, BioLegend), anti‐CD62L‐Alexa Fluor 700 (DREG‐56, BioLegend), anti‐CD11b‐PE/Dazzle 594 (ICRF44, BioLegend) and anti‐CD49d‐APC (9F10, BioLegend). The cells were incubated on ice for 30 min away from light. The red blood cells (RBCs) were then lysed with 2 ml cold isotonic solution (lysis buffer: 154 mM NH4Cl, 10 mM KHCO_3_ supplemented with 0·1 mM EDTA, pH 7·2), centrifuged for 5 min at 300× ***g*** at 4°, and washed with phosphate‐buffered saline (PBS) before being resuspended in 300 μl of cold PBS and subsequently analysed. The surface expression of CD203c, CD63, CD62L, CD11b and CD49d on basophils and the surface expression of CD62L, CD11b and CD49d on neutrophils were analysed by flow cytometry (Navios, Beckman Coulter).

### Flow cytometric analysis of cross‐linking of surface markers on basophils

Degranulation marker expression was analysed on basophils after cross‐linking of different surface markers. One hundred µl whole blood was incubated with the optimized concentration (data not shown) of unconjugated primary antibody towards CD203c (5 µg/ml, Abcam), CD62L (5 µg/ml, Abcam), CD11b (5 µg/ml, BioLegend) and CD49d (5 µg/ml, Abcam), diluted in RPMI, for 30 min at 4° or room temperature (RT). Thereafter, all tubes, except controls, were washed and incubated with unconjugated secondary antibodies diluted in RPMI at optimized concentrations (data not shown): 1 µg/ml anti‐mouse and 5 µg/ml anti‐rabbit antibodies (Abcam), at either 4° or room temperature for 25 min. Cells were then washed and stained using anti‐CD203c‐PE (Beckman coulter, Paris, France), which is a non‐competing antibody to the unconjugated primary anti‐CD203c antibody, and anti‐CD63‐FITC (Beckman Coulter) for 25 min at 4°. After that, the RBCs were lysed with 2 ml cold lysis buffer, and samples were centrifuged for 5 min at 300× ***g*** at 4° and then washed with PBS before being resuspended in 300  μl of cold PBS and subsequently analysed. The surface expression of CD203c and %CD63 on basophils was analysed by flow cytometry (Navios, Beckman Coulter). A different flow cytometry protocol and other blood donors were used to analyse the %CD63 on basophils and MFI CD203c for CD49d cross‐linking compared with the other surface markers.

### Cross‐linking of adhesion molecules or cytokine stimulation before cross‐linking of CD203c and detection of CD63 expression

The expression of degranulation markers was investigated after cross‐linking of adhesion markers or cytokine stimulation followed by CD203c cross‐linking. One hundred µl whole blood was incubated with unconjugated primary antibody towards CD62L (5 µg/ml, Abcam), CD11b (5 µg/ml, Abcam) or CD49d (5 µg/ml, Abcam), diluted in RPMI, for 30 min at 4°, followed by incubation with unconjugated secondary antibody (anti‐mouse (1 µg/ml, Abcam)), diluted in RPMI, at room temperature for 30 min.

In parallel, 100 µl whole blood was incubated with the following cytokines IL‐3 (10 ng/ml, Sigma‐Aldrich, St. Louis, Missouri, USA), IL‐33 (10 ng/ml, Thermo Fisher) or IL‐8 (100 ng/ml, R&D Systems, Minneapolis, Minnesota, USA), diluted in RPMI, at 37° for 30 min.

All tubes with either cross‐linked adhesion markers or cytokine‐stimulated cells were then incubated with unconjugated primary antibody towards CD203 (5 µg/ml, Abcam) for 30 min at 4°, followed by incubation for all tubes, except controls, with unconjugated secondary anti‐rabbit antibody (5 µg/ml, Abcam) at room temperature for 30 min. The tubes were then put on ice and incubated with anti‐CD203c‐PE (Beckman Coulter) and anti‐CD63‐FITC (Beckman Coulter) for 25 min and analysed using flow cytometry (Navios, Beckman Coulter).

### Flow cytometric analysis of adhesion molecules after stimulation with cytokines

The cells were stimulated with cytokines followed by detection of adhesion molecule expression. Fifty µl whole blood was incubated with IL‐3 (10 ng/ml, Sigma‐Aldrich), IL‐8 (100 ng/ml, R&D Systems) or IL‐33 (10 ng/ml, Thermo Fisher), diluted in RPMI, at 37° for 30 min, with RPMI as control. The stimulation was stopped by placing the cells on ice followed by incubation with the recommended concentration of antibodies for the basophil or the neutrophil panels for 30 min away from light. The RBCs were then lysed with 2 ml cold lysis buffer, and the samples were centrifuged for 5 min at 300× ***g*** at 4°, before washing with cold PBS and resuspended in 300 μl of cold PBS and subsequently analysed. The surface marker expression of CD62L, CD11b and CD49d on basophils and neutrophils was analysed by flow cytometry (Navios, Beckman Coulter).

### Cross‐linking of adhesion molecules or cytokine stimulation before basophil IgE‐dependent and IgE‐independent activation and detection of CD63 expression

Basophils were activated in an IgE‐dependent or IgE‐independent manner following prior cross‐linking of adhesion molecules or cytokine stimulation. One hundred µl whole blood was incubated with unconjugated primary antibody towards CD62L (5 µg/ml, Abcam), CD11b (5 µg/ml, Abcam) or CD49d (5 µg/ml, Abcam), diluted in RPMI, for 30 min at 4°, followed by incubation with 1 µg/ml unconjugated anti‐mouse secondary antibody (Abcam), diluted in RPMI, at room temperature for 30 min.

One hundred µl whole blood was incubated with the cytokines IL‐3 (10 ng/ml) or IL‐33 (10 ng/ml), diluted in RPMI, at 37° for 30 min.

All tubes with either cross‐linked adhesion markers or cytokine‐stimulated cells were then incubated with RPMI, 1 µg/ml anti‐IgE antibody (Beckman Coulter) or 5 × 10^−7 ^M fMLP for 30 min at 37°. The controls were only stimulated with RPMI, anti‐IgE antibody or fMLP. The tubes were then put on ice and incubated with anti‐CD203c‐PE (Beckman coulter) and anti‐CD63‐FITC (Beckman Coulter) for 25 min and analysed using flow cytometry (Navios, Beckman Coulter).

### Flow cytometric analysis and gating strategy

The flow cytometer was controlled daily using Flow‐Check (Beckman Coulter), and the stability of the compensations was examined daily using Flow‐Set (Beckman Coulter).

The absolute number of basophils and neutrophils was analysed. One hundred μl of whole blood was treated with the ImmunoPrep Reagent System (Beckman Coulter, USA) according to the manufacturer's instructions. Thereafter, the pretreated whole blood was mixed with 100 μl Flow‐Count beads (Beckman Coulter, USA), and the samples were analysed using flow cytometry, and the total number of leucocytes was counted.

**Figure 1:**
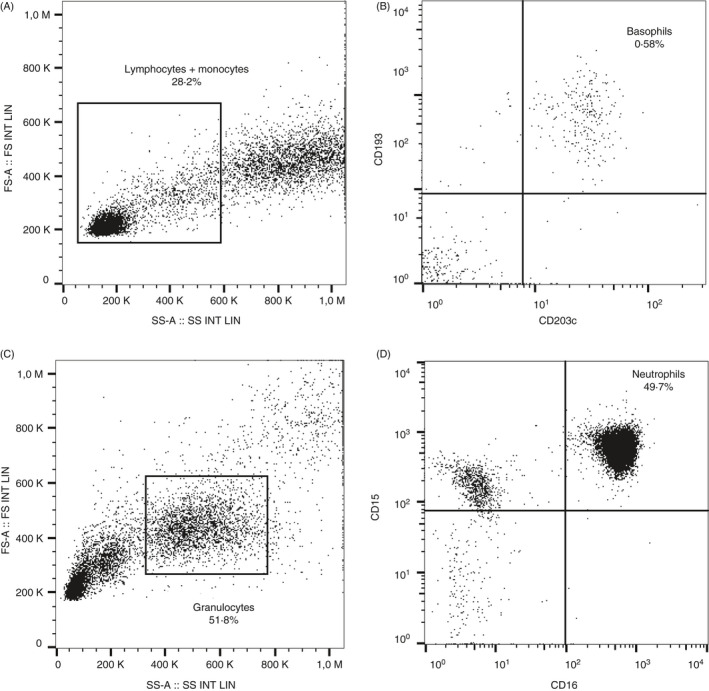
Gating strategies used for flow cytometry analysis of basophils and neutrophils. Gated (A) lymphocytes and monocytes were used to subsequently gate (B) CD193+ and CD203c+ basophils (percentage of basophils compared with total number of leucocytes). Gated (C) granulocytes were used to subsequently gate (D) CD15+ and CD16+ neutrophils (percentage of neutrophils compared with total number of leucocytes).

Further, basophils were identified as CD203c+, CD193+ (Figure 1A,B) and neutrophils as CD15+, CD16+ (Figure 1C,D). Mean fluorescence intensity (MFI) was used to evaluate activation of basophils and neutrophils by expression of CD62L, CD11b and CD49d. In addition, basophil activation was also measured as MFI for CD203c and per cent positive CD63 basophils (%CD63+). A cut‐off for a negative test was set to a baseline CD63 expression of approximately 2·5%.

### Statistical analysis

The statistical analysis and figures were generated in GraphPad Prism 7.0e (GraphPad Software, Inc., La Jolla, CA, USA). The histogram overlays were generated using FlowJo™ Software 10.6.2 (Becton Dickinson). Because the study population was not normally distributed, the results were presented as median and interquartile range and the significant differences between groups were analysed using the non‐parametric paired t‐test, the Wilcoxon test. Correlations were measured using the two‐tailed non‐parametric Spearman correlation test. A *P*‐value <0·05 was considered significant.

## RESULTS

### Comparison of surface marker expression on basophils and neutrophils before and after handling and flowing through a microfluidic chip

**Figure 2:**
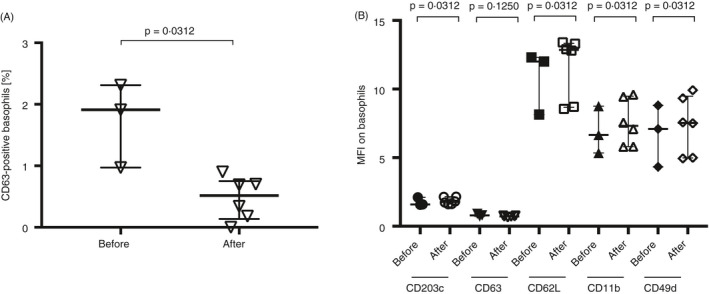
Surface marker expression on basophils, before inserted in the chip and after blood passed through a microfluidic chip. (A) %CD63+ (∇) basophils and (B) MFI for CD203c (○), CD63 (∇), CD62L (□), CD11b (∆) and CD49d (◊) before inserted in the chip and after passage through a microfluidic chip, where the blood sample from each donor (*n* = 3) was split and flown through two separate microfluidic chips (two chips per donor) (*n* = 6). Error bars are presented as median (interquartile range). A statistical significance was considered at a *P*‐value of <0·05. The Wilcoxon paired non‐parametric *t*‐test was used for statistical analysis.

Because of previous reports of high background activation of captured basophils in the microfluidic chip, the effect of handling and passage through the chip was investigated. This was analysed by measuring the changes in surface expression of CD203c, CD63, CD62L, CD11b and CD49d before and after flowing blood through a microfluidic chip. In addition, changes in neutrophil expression of CD62L and CD11b were analysed. The percentage of CD63 expression (%CD63+) on basophils was slightly lower after passage through the microfluidic chip than that before the procedure; however, the numbers were very low (Figure 2A). The MFI was slightly but significantly upregulated for CD203c, CD62L, CD11b and CD49d, but not for CD63 after blood was flown through the chip (Figure 2B).

In neutrophils, the MFI decreased significantly for CD62L expression after flowing through the microfluidic chip and the CD11b expression was slightly but not significantly upregulated (Figure S1).

### Expression of activation markers on basophils after cross‐linking of CD203c, CD62L, CD11b and CD49d

**Figure 3:**
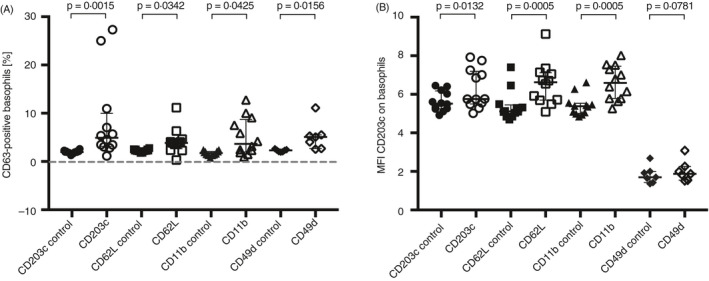
Cross‐linking of basophil surface markers and analysis of subsequent activation marker expression. Cross‐linking of CD203c (○), CD62L (□), CD11b (∆) or CD49d (◊) on basophils where (A) displays the %CD63+ basophils and (B) shows the MFI for CD203c on basophils. The controls are only incubated with the primary antibody. Error bars are presented as median (interquartile range). A statistical significance was considered at a *P*‐value of <0·05. The Wilcoxon paired non‐parametric *t*‐test was used for statistical analysis.

Considering the CD63 expression was not increased following passage through the microfluidic chip, the capture or cross‐linking of adhesion molecules was investigated as a possible mechanism for the background activation detected in basophils captured in the microfluidic chip. Surface markers CD203c, CD62L, CD11b (*n* = 12) and CD49d (*n* = 7) were cross‐linked with a primary and secondary antibody at different temperatures (4° or RT). The activation was not affected by different temperatures (data not shown); hence, data were pooled (Figure 3). The %CD63+ basophils after cross‐linking of surface markers were significantly increased compared with the control (only primary antibody) (Figure 3A). However, this activation was not comparable to the background level (median of 26%) seen in the microfluidic chip. The MFI for CD203c expression, when comparing control (only primary antibody) and cross‐linked surface molecules, was significantly higher for CD203c, CD62L and CD11b but not for CD49d (Figure 3B). There was a significant correlation between MFI CD203c expressions after cross‐linking of CD203c and CD62L, CD203c and CD11b, and CD11b and CD62L (Figure S2). The correlation could not be calculated for CD49d considering those experiments were obtained with a different set of blood donors.

### Effect of cross‐linking of CD62L, CD11b and CD49d followed by cross‐linking of CD203c on basophil activation

**Figure 4:**
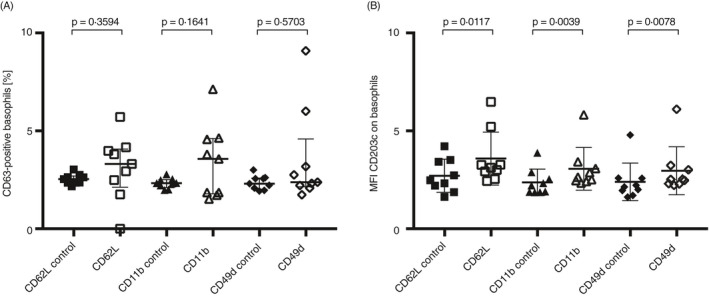
Sequential cross‐linking of basophil adhesion molecules followed by cross‐linking of CD203c and analysis of activation marker expression. Cross‐linking (*n* = 9) of CD62L (□), CD11b (∆) and CD49d (◊), followed by cross‐linking of CD203c and analysis of (A) %CD63+ basophils and (B) MFI for CD203c. Controls do not have the secondary antibody towards anti‐CD203c. Error bars are presented as median (interquartile range). A statistical significance was considered at a *P*‐value of <0·05. The Wilcoxon paired non‐parametric *t*‐test was used for statistical analysis.

As shown in previous experiments, cross‐linking of CD203c, CD62L, CD11b and CD49d increased the %CD63+ basophils and cross‐linking of CD203c, CD62L and CD11b increased MFI for CD203c (Figure 3A,B). Thus, we investigated whether sequential cross‐linking of CD62L, CD11b or CD49d followed by cross‐linking of CD203c could potentially increase the %CD63+ basophils further. However, no additional increase in %CD63+ basophils could be seen after cross‐linking of any of the markers compared with controls (Figure 4A). Yet, MFI for CD203c increased significantly as expected after cross‐linking with CD62L, CD11b and CD49d followed by CD203c cross‐linking (Figure 4B).

### Change in basophil surface marker expression after IL‐3, IL‐33 and IL‐8 stimulation

The effect of cytokines was investigated because of both their importance during basophil recruitment into the tissue, and the possibility of secretion from blood cells present in the microfluidic chip and impact on the captured basophils. The optimal cytokine concentration was obtained by dose–response titration and determined to be 10 ng/ml for IL‐3, 10 ng/ml for IL‐33 and 100 ng/ml for IL‐8 (*n* = 8) (Figure S2a–c) and used in the following experiments.

**Figure 5:**
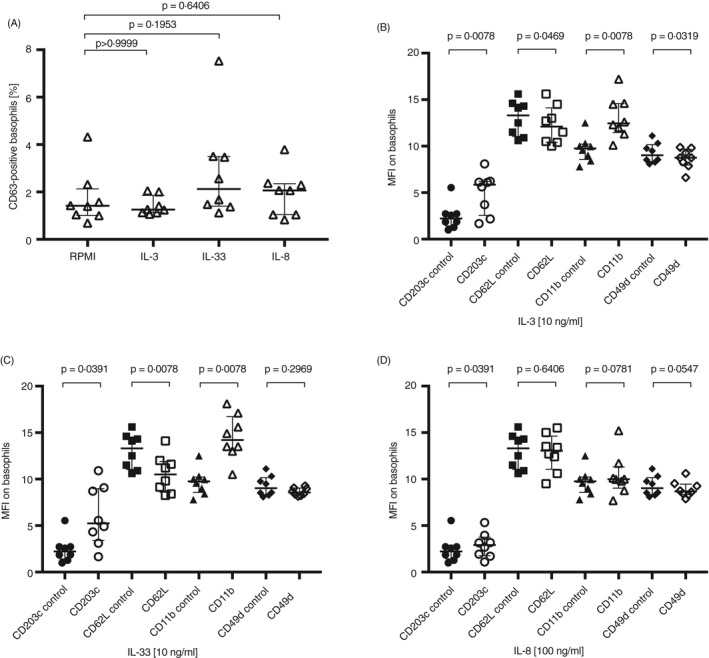
Cytokine stimulation of basophils and analysis of activation marker and adhesion molecule expression. Stimulation of basophils using cytokines IL‐3, IL‐33 and IL‐8. (A) %CD63+ basophils (*n* = 8) for the negative control (RPMI) and after IL‐3, IL‐33 and IL‐8 stimulation. Stimulation of basophils (*n* = 8) using (B) IL‐3, (C) IL‐33 and (D) IL‐8 and measure of MFI for CD203c (○), CD63 (∇), CD62L (□), CD11b (∆) and CD49d (◊) expression compared with the negative control (RPMI). Error bars are presented as median (interquartile range). A statistical significance was considered at a *P*‐value of <0·05. The Wilcoxon paired non‐parametric *t*‐test was used for statistical analysis.

None of the cytokines significantly affected the %CD63 expression (Figure 5A). IL‐3 significantly upregulated CD203c and CD11b on basophils, while CD62L and CD49d were significantly downregulated (Figure 5B). IL‐33 had similar effect on basophils and significantly upregulated CD203c and CD11b, while CD62L was significantly decreased, and the expression of CD49d was not significantly changed by IL‐33 (Figure 5C). IL‐8 only significantly upregulated CD203c on basophils (Figure 5D). Neutrophil stimulation with IL‐8 had the expected results with significant CD62L shedding and CD11b and CD49d upregulation (Figure S3).

### Effect of cytokine stimulation followed by CD203c cross‐linking on basophil activation

As shown earlier, sequential cross‐linking of adhesion markers followed by cross‐linking of CD203c did not increase %CD63+ basophils. However, here we tested whether sequential cytokine stimulation followed by cross‐linking of CD203c could potentially have additional effects on %CD63 or CD203c expression.

**Figure 6:**
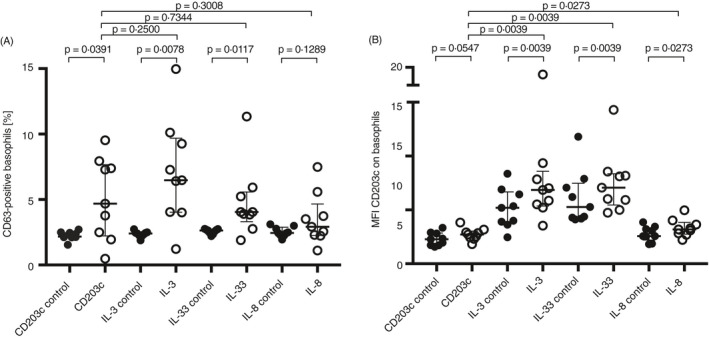
Sequential stimulation of basophils with cytokines followed by cross‐linking of CD203c and detection of activation markers. Stimulation with IL‐3, IL‐33 or IL‐8 (*n* = 9) followed by cross‐linking of CD203c and analysis of (A) %CD63+ basophils and (B) MFI for CD203c. Controls do not have the secondary antibody towards anti‐CD203c. Error bars are presented as median (interquartile range). A statistical significance was considered at a *P*‐value of <0·05. The Wilcoxon paired non‐parametric *t*‐test was used for statistical analysis.

CD203c cross‐linking did significantly upregulate %CD63+ basophils, but stimulation with the cytokines IL‐3, IL‐33 or IL‐8 followed by CD203c cross‐linking did not further significantly potentiate the upregulation of %CD63+ basophils compared with the controls (primary anti‐CD203c antibody, no cross‐linking) (Figure 6A). However, cytokine stimulation followed by CD203c cross‐linking significantly increased the expression of CD203c on basophils. There was a significant increase in CD203c MFI for IL‐3, IL‐33 and IL‐8. Cytokine stimulation alone, without CD203c cross‐linkage, did also significantly increase MFI for CD203c, while CD203c cross‐linking alone, without cytokine stimulation, did not significantly increase MFI for CD203c (Figure 6B).

### IgE‐dependent and IgE‐independent basophil activation after adhesion molecule cross‐linking or cytokine stimulation

**Figure 7:**
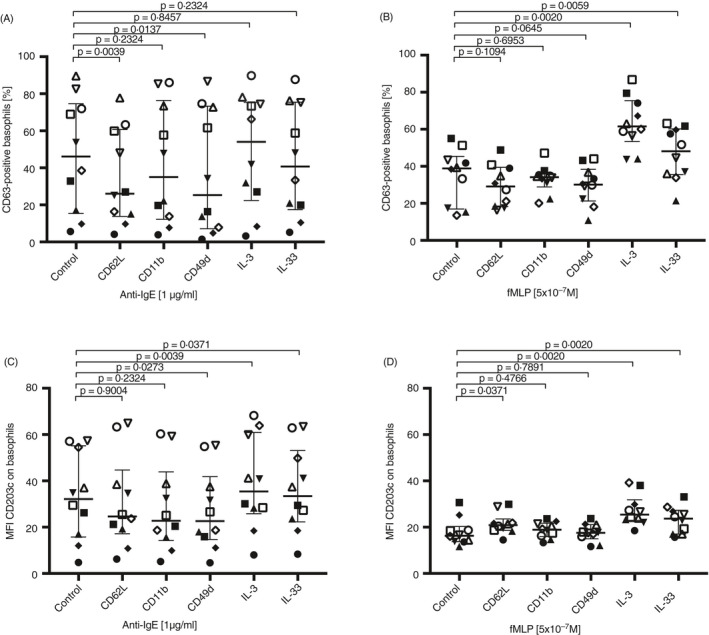
Sequential stimulation of basophils with cytokines or cross‐linking of adhesion molecules followed by IgE‐ and non‐IgE‐mediated degranulation. Activation of basophils (*n* = 10) using (A) anti‐IgE antibody or (B) fMLP followed by detection of %CD63+ or (C) anti‐IgE antibody or (D) fMLP activation followed by detection of MFI CD203c, after cross‐linking of CD62L, CD11b or CD49d or stimulation with IL‐3 or IL‐33. Each donor is identified with a unique symbol. Error bars are presented as median (interquartile range). A statistical significance was considered at a *P*‐value of <0·05. The Wilcoxon paired non‐parametric *t*‐test was used for statistical analysis.

**Figure 8:**
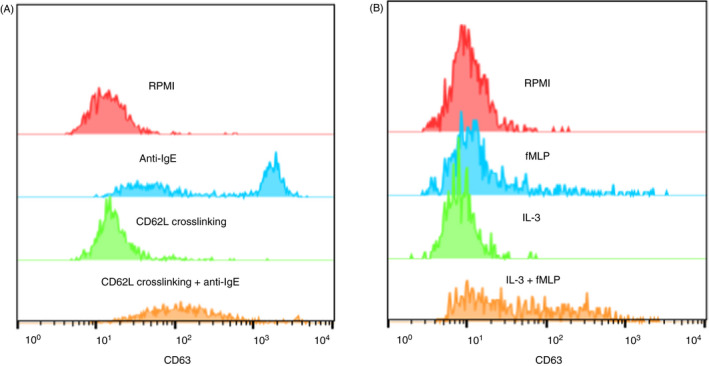
Histogram overlays for %CD63 expression on basophils after cross‐linking of CD62L and IL‐3 stimulation followed by IgE‐ and non‐IgE‐mediated degranulation. (A) RPMI or anti‐IgE activation alone compared with sequential CD62L cross‐linking followed by anti‐IgE activation. (B) RPMI or fMLP activation alone compared with sequential IL‐3 stimulation followed by fMLP activation.

Both adhesion and cytokine stimulation are important factors for basophil recruitment into the tissue during inflammation, and once in tissue, the cell can become activated. The effect of IgE‐dependent and IgE‐independent stimulation on basophil activation was studied following either cross‐linking of adhesion molecules or cytokine stimulation. Sequential cross‐linking of CD62L or CD49d followed by stimulation using anti‐IgE antibody resulted in a significantly lower basophil activation compared with activation with anti‐IgE without prior cross‐linking (Figure 7A). The median %CD63 expression was 46·2% (15·4–74·6%) for the control (anti‐IgE alone), 26·1% (13·8–60·7%) for CD62L cross‐linking followed by anti‐IgE activation and 25·3% (7·11–73·3%) for CD49d cross‐linking followed by anti‐IgE activation (Figure 7A). This has been visualized in histogram overlays that show CD62L cross‐linking and IgE‐dependent activation compared with controls (Figure 8A). Sequential cytokine stimulation with IL‐3 or IL‐33 followed by fMLP activation significantly increased the %CD63 expression compared with fMLP activation alone (Figure 7B). The median %CD63 expression was 38·9% (16·9–45·4%) for the control (fMLP alone), 61·5% (53·4–75·5%) for IL‐3 stimulation followed by fMLP activation and 48·1% (35·5–60·2%) for IL‐33 stimulation followed by fMLP activation (Figure 7B). This has been visualized in histogram overlays that show IL‐3 stimulation and IgE‐independent activation compared with controls (Figure 8B). The MFI for CD203c changed after anti‐IgE activation and included a significant decrease from CD49d cross‐linking and a significant increase from IL‐3 and IL‐33 stimulation before anti‐IgE activation, compared with only anti‐IgE activation (Figure 7C). The MFI for CD203c changed after fMLP activation where a significant increase was detected after CD62L cross‐linking and from IL‐3 and IL‐33 stimulation followed by fMLP activation compared with fMLP activation alone (Figure 7D).

## DISCUSSION

In this study, we demonstrate that handling of the blood and passage through the microfluidic chip do not impact the CD63 expression on basophils. A significant increase in %CD63 expression is observed following CD203c and adhesion molecule cross‐linking. Moreover, cross‐linking of CD62L and CD49d has a regulatory effect on subsequent IgE‐dependent degranulation by anti‐IgE, while IL‐3 and IL‐33 exposure increases the subsequent IgE‐independent degranulation by fMLP.

The background expression of CD63 on captured basophils is a challenge in miBAT, which limits the analysis range. In miBAT, blood is flown through the chip using a syringe pump and involves handling steps not included in the flow cytometry analysis, which can potentially affect the cells and cause background activation. Platelet or leucocyte activation has previously been shown to occur when flowing through different types of tubing systems in, for example, cardiopulmonary bypass^20^ and in an artificial stenosis model.^21^ However, in our study, the CD63 expression on basophils is not affected by passage of blood through the microfluidic chip and consequently cannot be accounted for the background activation. As a control, neutrophils significantly shed CD62L, which has previously also been seen after contact with polydimethylsiloxane (PDMS).^22^

A possible cause for the elevated CD63 expression in miBAT is the capture of the basophils in the microfluidic chip using an immobilized anti‐CD203c antibody. Additional factors can be that basophil adhesion molecules bind adsorbed proteins from blood such as fibronectin in the miBAT. Indeed, it has been shown that PDMS can adsorb proteins such as fibronectin from blood.^22^ Fibronectin is a ligand for both CD11b and CD49d^23^ and can potentially cause interactions between basophils and the adsorbed proteins.

Cross‐linking of adhesion molecules is considered to occur in vivo during the recruitment process when the formation of adhesion molecule clusters amplifies the overall avidity.^24^ CD62L is of interest for its role in basophil adhesion and its unique stable expression on basophils.^13^ Indeed, cross‐linking of CD203c and that of the adhesion molecules CD62L, CD11b and CD49d show a significant basophil activation judged by %CD63 expression. These experiments were performed at different temperatures (4° or RT) to investigate the possibility of a temperature‐dependent effect. However, temperature does not have an impact on CD63 expression; hence, a change in temperature during chip experiments will not reduce the background signal. After cross‐linking of CD203c, CD62L and CD11b, respectively, we find a strong correlation of the CD203c MFI values between respective cross‐linking. This implies that cross‐linking of CD203c, CD62L and CD11b has a similar propensity to cause piecemeal degranulation. On the other hand, there is no correlation between cross‐linkage of the three markers and anaphylactic degranulation (CD63 expression).

We also explore whether the increase in CD203c MFI impacts the degranulation (CD63 expression) when cross‐linking occurred in sequence, that is first cross‐ linking of adhesion molecules followed by cross‐linking of CD203c. However, we observe no additional activation, which indicates that the increased CD203c expression is insufficient to affect the CD63 expression when cross‐linked. Together, these data demonstrate that a qualitative difference exists how engagement of these receptors impacts the degranulation process in basophils.

All cytokines (IL‐3, IL‐33 and IL‐8) significantly upregulate CD203c. IL‐3 and IL‐33 have the expected effect and upregulate CD11b on basophils, while in contrast, CD62L is slightly but significantly shed from basophils. Cytokine interactions are important for basophil recruitment into the tissue but could also potentially influence captured basophils in miBAT. IL‐3 is present in the stimulation buffer used in miBAT,^11^ and there is a possibility that a variety of cytokines are secreted from other blood cells also present in the chip.^22^ Hence, we studied the effect of three cytokines on the expression of adhesion markers on basophils. CD62L has been found to be quite stable on the basophil surface regardless of stimuli.^13^ The observation that IL‐33 has a more pronounced effect on CD62L shedding than IL‐3 and fMLP is of special interest as IL‐33 is secreted by endothelial cells^16^ and mast cells^25^ and is involved in the recruitment of basophils into the inflammatory foci. Considering that IL‐33 is mainly found in tissues, we speculate that IL‐33‐dependent CD62L shedding is more likely to occur at extravascular sites. In tissue, CD62L has the potential to interact with ligands such as proteoglycans,^26^ which might be regulated by CD62L shedding via IL‐33. This suggests a regulatory role for IL‐33 in basophil adhesion, transmigration and degranulation.

We also explored the effect of sequential cytokine stimulation and CD203c cross‐linking. The rational was to investigate whether cytokine‐induced upregulation of CD203c could affect the degranulation after subsequent CD203c cross‐linking. However, stimulation with IL‐3 and IL‐33 followed by CD203c cross‐linking does not further potentiate the anaphylactic degranulation judged by CD63 expression.

Based on our observations that adhesion molecule cross‐linking and cytokine exposure alter the basophil state of activation, we studied how both these factors affect basophil degranulation processes, via IgE‐dependent and IgE‐independent stimulation. This is of interest in vivo and in miBAT as we stimulate the captured basophils with allergens (IgE‐dependent activation) in the chip. Cross‐linking of basophil adhesion molecules or incubation with IL‐3 or IL‐33 was followed by activation via either anti‐IgE antibodies or fMLP. Cross‐linking of CD62L or CD49d followed by IgE‐dependent degranulation shows a significant decrease in %CD63+ basophils compared with IgE‐dependent degranulation alone, which implies a regulatory role for CD62L‐ and CD49d‐dependent adhesion. This was not the case for CD11b cross‐linking, which indicates that the effect is adhesion pathway‐specific. This regulatory effect has previously been reported in asthmatic patients where CD49d cross‐linking decreased histamine release after anti‐IgE antibody activation, and it was also stated that cross‐linking of CD49d equalled binding to fibronectin.^27^

The regulatory effect of CD62L cross‐linking on IgE‐dependent degranulation has not previously been shown and could possibly play a protective role by minimizing the risk of IgE‐dependent degranulation before reaching the site of inflammation in tissue where the receptor could be shed upon IL‐33 exposure. This observation is also of importance for the miBAT technique as a high background in combination with a lower activation with anti‐FcεRI and relevant allergens in chip (miBAT) has been observed compared with flow cytometry‐based BAT.^11^ Cross‐linking of CD203c and activation would therefore also be essential conditions to investigate further and IgE‐independent activation in miBAT. In contrast to adhesion molecule cross‐linking, IL‐3 and IL‐33 exposure followed by IgE‐independent degranulation via fMLP increases the CD63 expression. The bacterial peptide fMLP is known to cause anaphylactic degranulation in basophils.^4^, ^5^ IL‐3 has been reported to increase the effect of IgE‐independent anaphylactic degranulation in basophils.^28^ However, to our knowledge, IL‐33 has not previously been shown to have the same impact as IL‐3. IL‐33 has been described to increase the effect of IgE‐dependent degranulation in mast cells,^29^ is involved in recruitment of basophils in allergic disease^25^ and can be produced in response to, for example, parasite and bacterial infections.^30^ It is therefore plausible that IL‐33 influences IgE‐independent degranulation in basophils.

A limitation of this study was the use of two different flow cytometry protocols when analysing the cross‐linking of adhesion molecules. CD49d cross‐linking was performed at a later stage than the other surface markers and was therefore also analysed using blood from different blood donors. Hence, the MFI values for CD49d cannot be compared with the MFI for the other surface markers. However, it does not affect the interpretation of the results. Another limitation is that the experiments do not mimic all complex steps that occur in vivo during adhesion and degranulation. The experiments focus on separate parts of the process such as the effect of CD62L cross‐linking on IgE‐dependent degranulation not taking other potential interactions into consideration. However, this could also be regarded as an advantage as it allows us to detect the effect of individual steps in the adhesion and degranulation process for basophils. One should consider the consequence of the limited number of experiments included, and more studies are needed to gain more knowledge.

We identify cross‐linking of several surface markers as potential factors in the background activation found in miBAT. Engagement of CD62L and CD49d has a regulatory effect on IgE‐dependent activation and degranulation but not on IgE‐independent activation. We report that IL‐33 has a slightly greater effect on CD62L shedding than, for example, IL‐3 and that CD62L cross‐linking decreases IgE‐dependent basophil activation. We therefore speculate that a protective mechanism for the degranulation process exists during the transmigration process involving a dynamic regulation of adhesion molecule expression, ligand binding and cytokine exposure. We also show an increased IgE‐independent basophil activation after IL‐3 and IL‐33 stimulation, which warrants additional studies combining cross‐linking of adhesion molecules to mimic different scenarios in vivo and in miBAT. Together, our data denote that basophil regulatory mechanisms that are operational both in the microfluidic chip environment and in vivo might impact basophil degranulation procedures such as anaphylactic degranulation and piecemeal degranulation and hence the basophil immune regulatory function.

## CONFLICT OF INTEREST

The authors have no commercial or financial conflict of interest.

## AUTHOR CONTRIBUTION

Frida Kalm, Joachim Lundahl and Anna Nopp designed the study. Frida Kalm performed all experiments with support from Ladan Mansouri. Frida Kalm wrote the paper with input from Joachim Lundahl, Aman Russom, Ladan Mansouri and Anna Nopp.

## FUNDING INFORMATION

This study was supported by grants from VINNOVA, Vårdalstiftelsen, Astma‐ och Allergiförbundet, Svenska Föreningen för Allergologi, Ellen, Walter and Lennart Hesselman Foundation for Scientific Research, Cancer och Allergifonden, HMT KTH‐SLL and Karolinska Institutet. The funding sources had no involvement in the conduct of the research nor in the preparation of the manuscript.

## ETHICAL APPROVAL

The study was approved by the regional ethics committee in Stockholm, Sweden (Dnr. 2014/1630–31/4). Informed consent was obtained from all study participants.

## Supporting information


**Figure S1.** MFI for adhesion molecules on neutrophils. MFI for surface marker expression of CD62L (□) and CD11b (∆) on neutrophils, before (*n* = 3) and after blood has been flown through a microfluidic chip (*n* = 6). Error bars are presented as median (interquartile range). A statistical significance was considered at a *P*‐value of <0·05. The Wilcoxon paired non‐parametric *t*‐test was used for statistical analysis.

**Figure S2.** Correlation between MFI CD203c on basophils after crosslinking of surface markers. Correlations (*n* = 12) of MFI for CD203c after crosslinking with (a) CD62L and CD203c (*r* = 0·8811, *P* = 0·0003), (b) CD11b and CD203c (*r* = 0·8091, *P* = 0·0022) and (c) CD62L and CD11b (*r* = 0·9072, *P* = 0·0001). Correlations were measured using the two‐tailed non‐parametric Spearman correlation test. A *P* value of <0·05 was considered significant.

**Figure S3.** Cytokine stimulation and detection of MFI for adhesion molecules on basophils and neutrophils. Stimulation and measure of MFI for (a) CD203c (○) on basophils using cytokines IL‐3, IL‐33 and IL‐8, for (b) CD62L (□) and CD11b (∆) on basophils using cytokines IL‐8 and IL‐33 and for (c) CD62L (□) and CD11b (∆) on neutrophils using cytokines IL‐8 and IL‐33, at 1, 10 and 100 ng/ml. Error bars are presented as median (interquartile range). A statistical significance was considered at a *P*‐value of <0·05. The Wilcoxon paired non‐parametric t‐test was used for statistical analysis.

**Figure S4.** Cytokine stimulation of neutrophils and detection of MFI for adhesion molecules. Stimulation of neutrophils (*n* = 8) and MFI measurements for expression of CD62L (□), CD11b (∆) and CD49d (◊) compared to the negative control (RPMI) using cytokines IL‐3, IL‐8 and IL‐33. Error bars are presented as median (interquartile range). A statistical significance was considered at a *P*‐value of <0·05. The Wilcoxon paired non‐parametric *t*‐test was used for statistical analysis.

## Data Availability

The data that support the findings of this study are available from the corresponding author upon reasonable request.
