# Supplementary figures and images for: Adhesion molecule cross‐linking and cytokine exposure modulate IgE‐ and non‐IgE‐dependent basophil activation
Source: Immunology. 2020 Oct 29;162(1):92–104. doi: 10.1111/imm.13268 (PMC7730031; doi:10.1111/imm.13268)

Figure S1

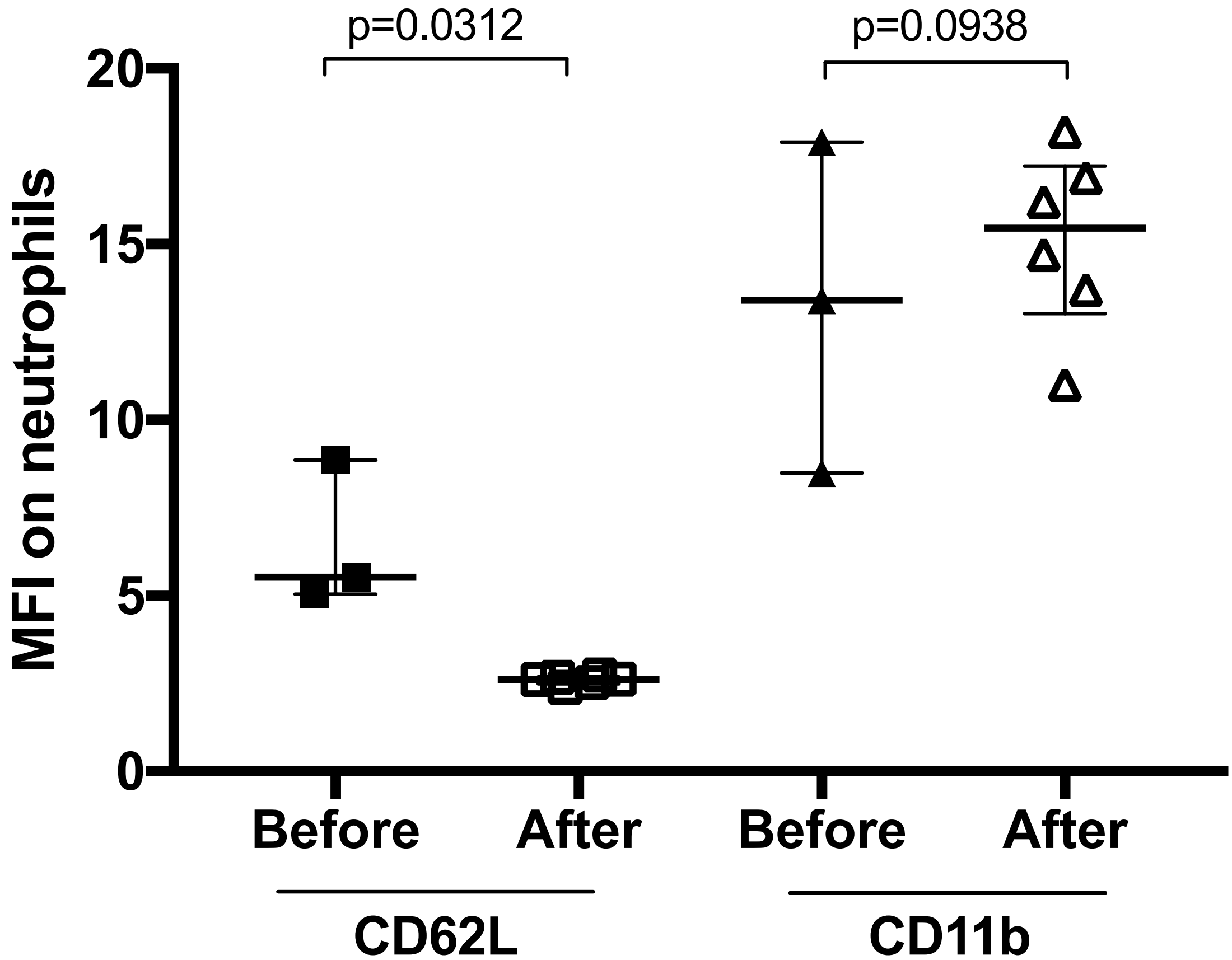

Supplement: Supplementary file 1 — Figure S1. MFI for adhesion molecules on neutrophils. MFI for surface marker expression of CD62L (□) and CD11b (∆) on neutrophils, before (n = 3) and after blood has been flown through a microfluidic chip (n = 6). Error bars are presented as median (interquartile range). A statistical significance was considered at a P‐value of <0·05. The Wilcoxon paired non‐parametric t‐test was used for statistical analysis. [file IMM-162-92-s001.pdf]

Figure S2

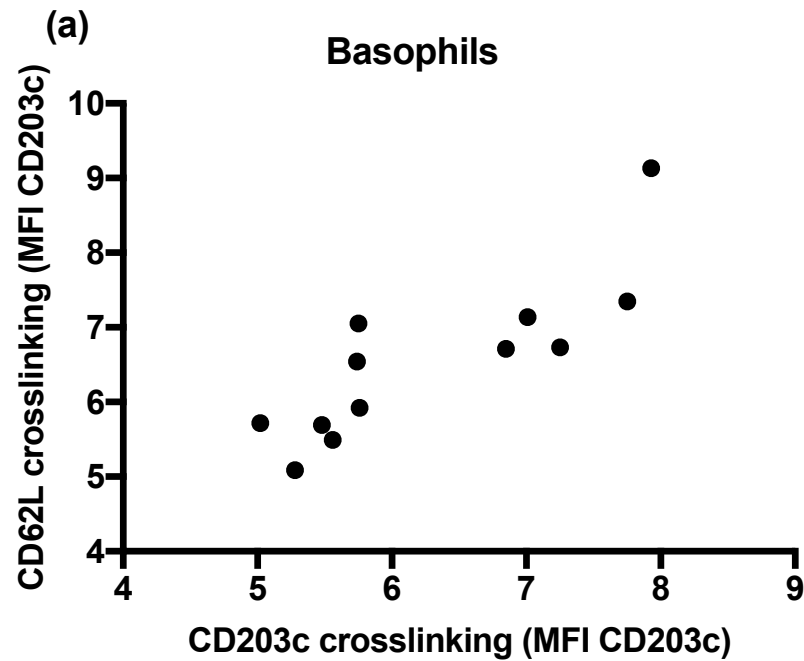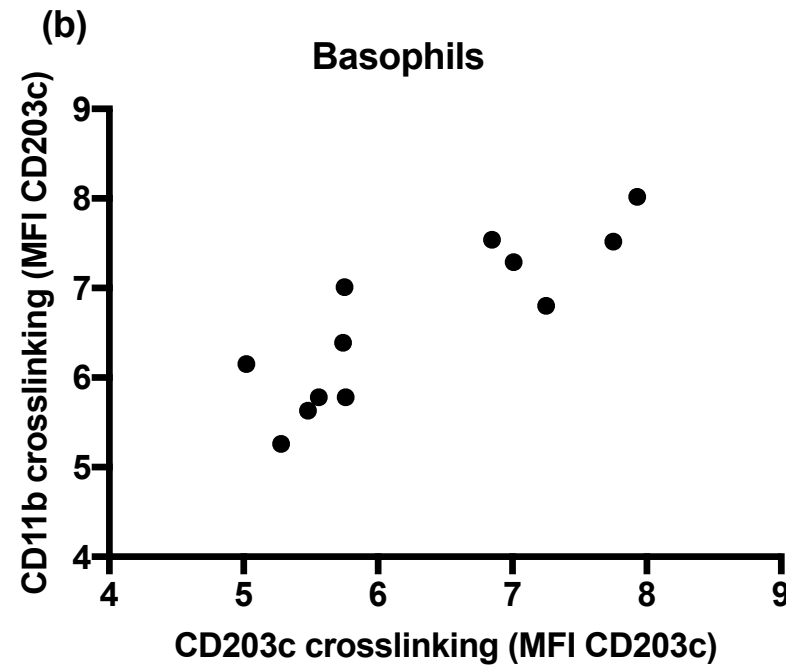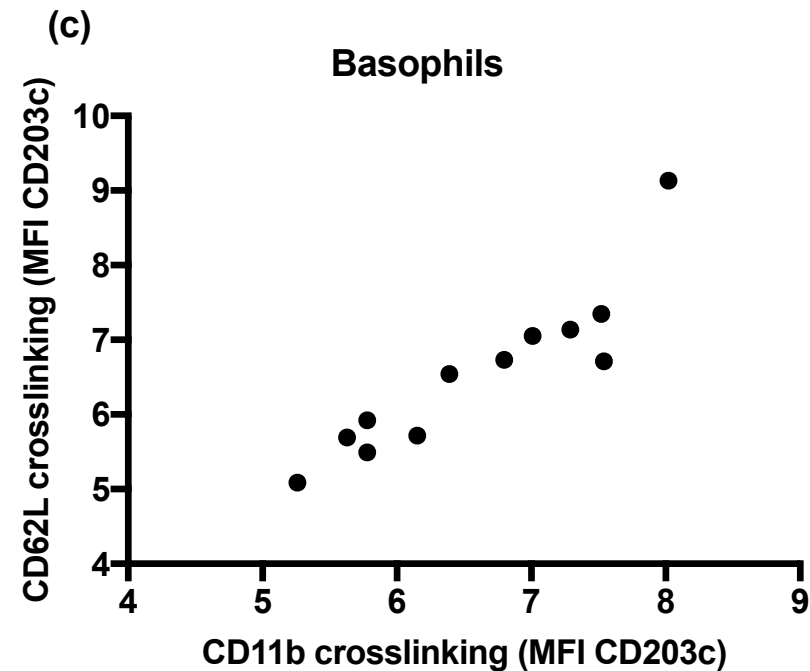

Supplement: Supplementary file 2 — Figure S2. Correlation between MFI CD203c on basophils after crosslinking of surface markers. Correlations (n = 12) of MFI for CD203c after crosslinking with (a) CD62L and CD203c (r = 0·8811, P = 0·0003), (b) CD11b and CD203c (r = 0·8091, P = 0·0022) and (c) CD62L and CD11b (r = 0·9072, P = 0·0001). Correlations were measured using the two‐tailed non‐parametric Spearman correlation test. A P value of <0·05 was considered significant. [file IMM-162-92-s002.pdf]

Figure S3

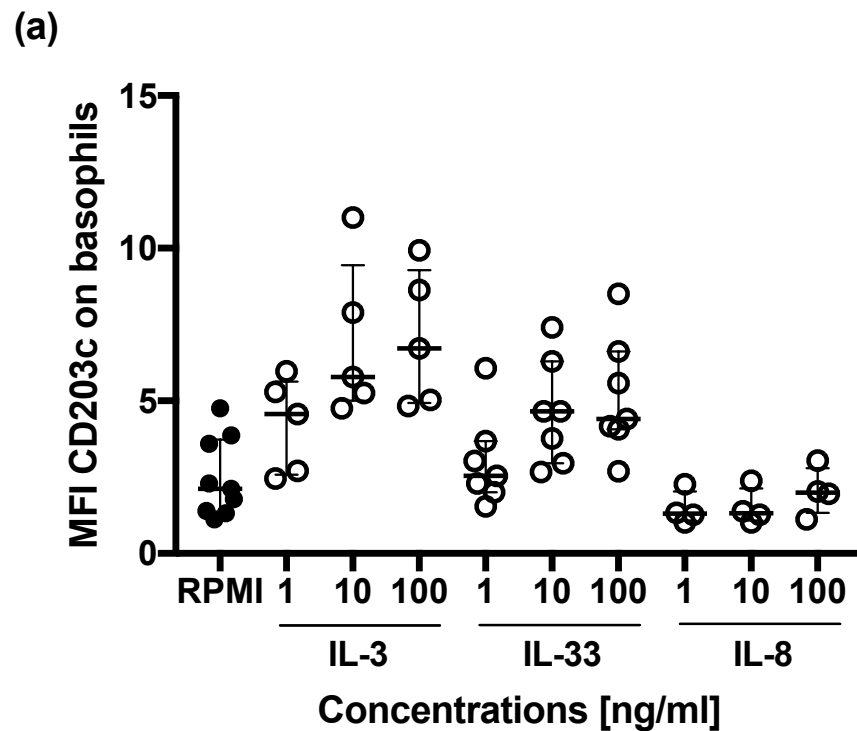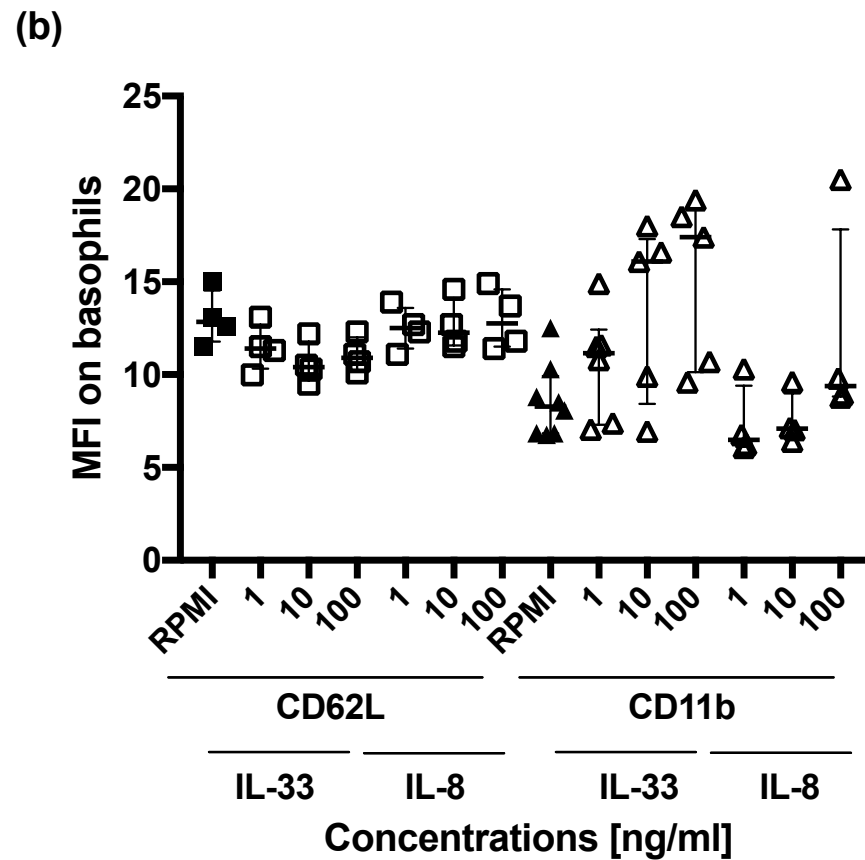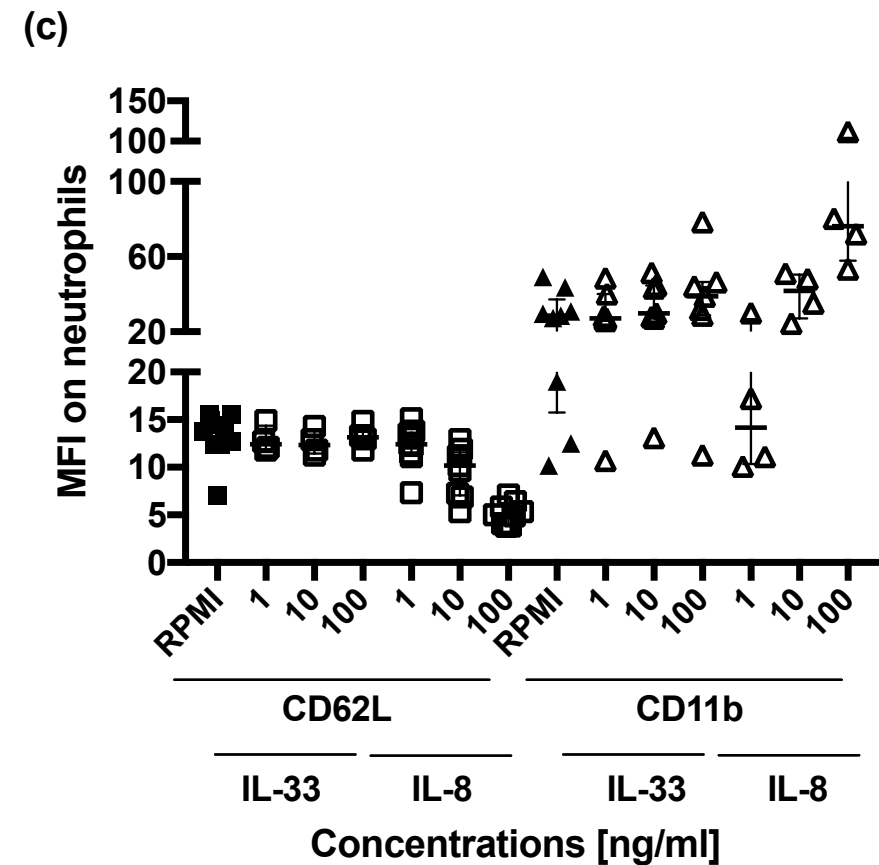

Supplement: Supplementary file 3 — Figure S3. Cytokine stimulation and detection of MFI for adhesion molecules on basophils and neutrophils. Stimulation and measure of MFI for (a) CD203c (○) on basophils using cytokines IL‐3, IL‐33 and IL‐8, for (b) CD62L (□) and CD11b (∆) on basophils using cytokines IL‐8 and IL‐33 and for (c) CD62L (□) and CD11b (∆) on neutrophils using cytokines IL‐8 and IL‐33, at 1, 10 and 100 ng/ml. Error bars are presented as median (interquartile range). A statistical significance was considered at a P‐value of <0·05. The Wilcoxon paired non‐parametric t‐test was used for statistical analysis. [file IMM-162-92-s003.pdf]

Figure S4

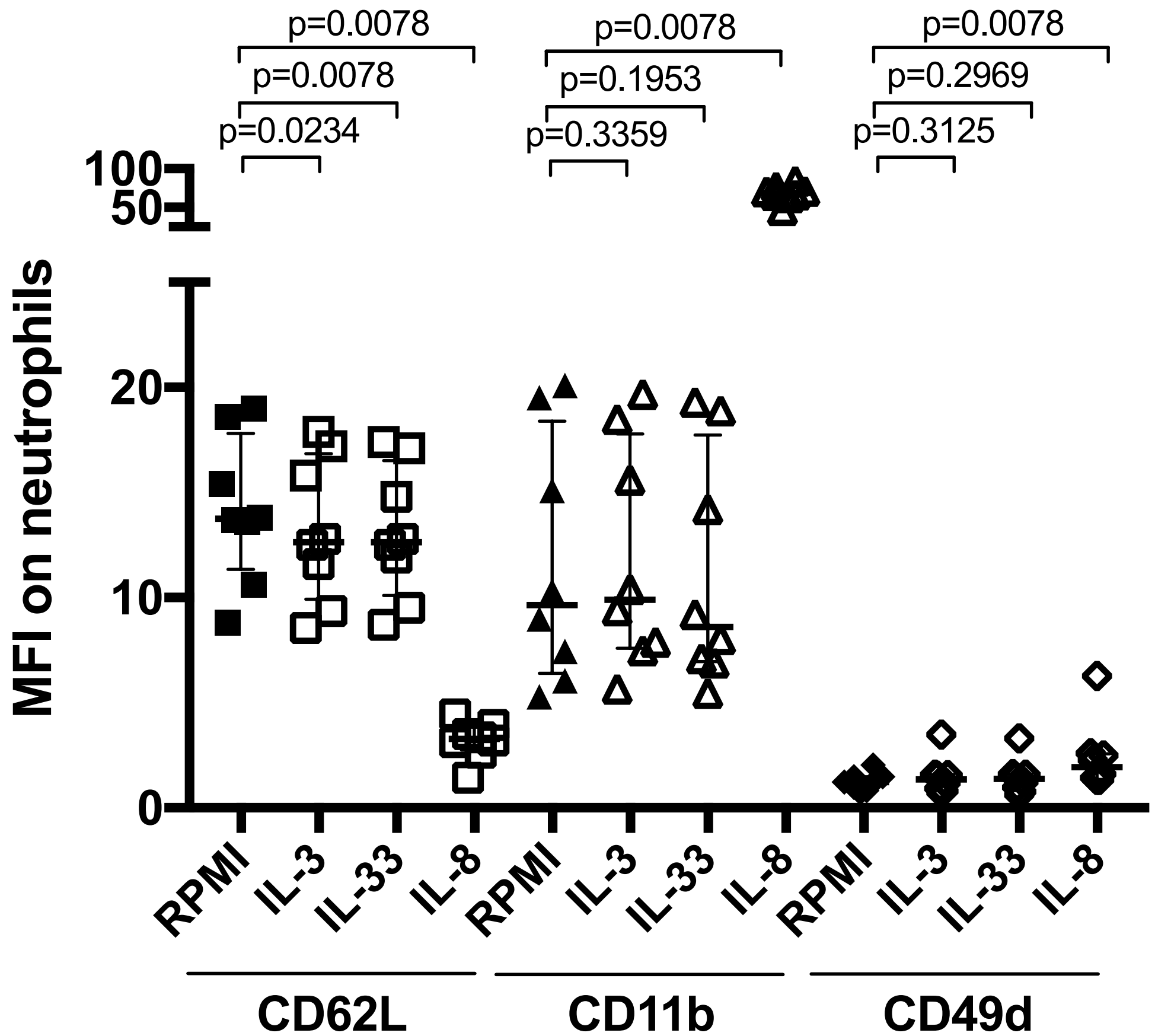

Supplement: Supplementary file 4 — Figure S4. Cytokine stimulation of neutrophils and detection of MFI for adhesion molecules. Stimulation of neutrophils (n = 8) and MFI measurements for expression of CD62L (□), CD11b (∆) and CD49d (◊) compared to the negative control (RPMI) using cytokines IL‐3, IL‐8 and IL‐33. Error bars are presented as median (interquartile range). A statistical significance was considered at a P‐value of <0·05. The Wilcoxon paired non‐parametric t‐test was used for statistical analysis. [file IMM-162-92-s004.pdf]
